# Genome-wide identification and analysis of *miR396* family members and their target GRF genes in rubber tree (*Hevea brasiliensis*)

**DOI:** 10.1186/s12864-025-12156-x

**Published:** 2025-10-31

**Authors:** Mingming Liu, Shenghe Zhao, Jiameng Wang, Zhihua Tu, Jinhui Chen

**Affiliations:** 1 School of Breeding and Multiplication (Sanya Institute of Breeding and Multiplication), School of Tropical Agriculture and Forestry, Hainan University, Sanya, 572019 China; 2 Engineering Research Center of Rare and Precious Tree Species in Hainan Province, School of Tropical Agriculture and Forestry, Hainan University, Haikou, 570228 China

**Keywords:** *miR396* family, Phylogenetic evolution, Rubber tree, HbrGRFs

## Abstract

**Background:**

MicroRNAs (miRNAs) are key regulators of gene expression as they play crucial roles at the post-transcriptional level. In particular, the *miR319*-GRF module is an important gene regulatory network in plants, extensively involved in processes such as plant growth and development. Although *miR396* is one of the most conserved miRNA families, its role in rubber trees remains poorly understood. In this study, bioinformatics analysis, including target prediction, was performed to reveal the evolutionary and expression patterns of the *Hbr-miR396* family members.

**Results:**

A total of six *Hbr-miR396* members were identified, distributed across four chromosomes. Secondary structure analysis revealed that the precursor sequences of the six *Hbr-miR396* members could form a typical stem-loop (hairpin) structure. Sequence analysis show that the members of the *Hbr-miR396* family form three mature sequences. Furthermore, phylogenetic analysis demonstrated that the *Hbr-miR396* family members are closely related to those from cassava. Eight members of the growth regulatory factor (GRF) family were predicted as potential targets of *Hbr-miR396*. The dual-luciferase assays also confirmed that *Hbr-miR396b* strongly inhibited the expression of HbrGRF3. Expression analysis of the HbrGRF targets in different tissues revealed that HbrGRFs are mainly expressed in the cambium and flowers. Therefore, *Hbr-miR396* may potentially regulate growth and floral organ development in rubber trees by targeting HbrGRFs.

**Conclusions:**

The data presented in this study offer valuable insights into the functional and molecular regulatory mechanisms of the *miR396*-GRFs module in rubber tree growth and development, laying a foundation for further investigation into its biological roles in enhancing both rubber production and timber quality.

**Supplementary Information:**

The online version contains supplementary material available at 10.1186/s12864-025-12156-x.

## Background

MicroRNAs (miRNAs) are endogenous RNA molecules of approximately 22 nucleotides. They play important regulatory roles in plants and animals by targeting mRNAs for cleavage or translational repression [1]. They were first discovered in the nematode *Caenorhabditis elegans* in 1993 [2]. However, the first miRNA in plants was reported in 2002, when miRNAs were discovered in *Arabidopsis thaliana* [3]. Since then, with the development and application of high-throughput sequencing [4], an increasing number of miRNAs have been identified in various plant species, such as in radish [5], tea plant [6], strawberry [7], and so on. To date, miRNAs from 271 species have been studied [8].

The miRNAs are regarded as key regulators of gene expression because they play a crucial role at the post-transcriptional level by blocking translation or promoting the degradation of target mRNAs [1]. miRNAs are involved in various biological processes, including plant growth and development [9–12], and responses to biotic and abiotic stresses [6, 13, 14]. Among them, the *miR396* family is one of the well-conserved families in plants, which was first identified using a devised computational procedure, and the expression of two members, *miR396a* and *miR396b*, was validated in *Arabidopsis thaliana* through Northern blot analysis in 2004 [14]. In the *A. thaliana* genome, there are two *miR396-*encoding loci: athMIR396a and athMIR396b [14]. Members of the *miR396* family extensively target the growth-regulating factor (GRF) genes to mediate plant growth and development [15–18]. In *Arabidopsis*, *e*ctopic expression of *miR396* suppresses GRF target gene expression and alters leaf growth [19]. Further, *miR396* affects the growth and development of lettuce leaves by inhibiting the expression of plant-specific GRFs [20]. In *A. thaliana*, compared to wild-type plants, the overexpression of *miR396* weakens the regulatory effect of GRFs, leading to abnormal root phenotype development and severely impairing root growth [9]. Therefore, the *miR396*-GRF module plays an important regulatory role in root organ development in plants. The *miR396*-GRF module is also involved in the development of rice floral organs, when the *miR396d* overexpression had resulted in the production of more petals as compared to the wild-type plants [12]. Additionally, studies have shown that the heterologous expression of *miR396c* in *Populus* results in altered flower development and a significant decrease in the expression of the *NtGRF* gene in tobacco [10]. In addition, studies have shown that rice *Osa-miR396c* enhances salt tolerance in the transgenic plants by improving water retention, chlorophyll content, membrane integrity, and Na⁺ exclusion [21]. Recent research has also revealed that the *miR396b*-GRF6 module enhances rice salt tolerance by reducing ROS accumulation and increasing ROS-scavenging enzyme activity [22]. Recent studies have shown that *miR396* also plays a critical role in the resistance of alfalfa against insects, as down-regulation of *miR396* expression in MIM396 transgenic plants enhances their resistance to *Spodoptera litura*, primarily by increasing the lignin content by regulating the related biosynthetic pathways [23]. These studies, thus have revealed the functional mechanisms of the *miR396*-GRF module.

The rubber tree (*Hevea brasiliensis* Muell. Arg.), a perennial species of the *Euphorbiaceae* family, is the primary source of natural rubber [24]. Rubber trees are used to produce a variety of products, such as pulpwood for papermaking, rubber-based composite boards, furniture, and fine woodworking products [25–27]. The economic importance of the rubber tree and its growing demand have promoted its extensive domestication. Given the increasing economic and application value of the rubber tree, research on its miRNAs has been reported a few times, most of which is related to high-throughput sequencing [28, 29]. However, studies on the systematic analysis of the *miR396* family members in the rubber tree and the prediction of their target genes have remained limited.

This study focused on the rubber tree *Hbr-miR396* family, by conducting a systematic analysis of its precursor and mature sequences. Furthermore, target gene prediction was performed by using bioinformatics tools. The interaction between *Hbr-miR396b* and its target genes was further validated through a dual-luciferase reporter assay. The results of this study provide a theoretical support for further understanding the potential role of *Hbr-miR396* family members in the growth and development of rubber trees.

## Results

### Sequence analysis and chromosomal localization of the *Hbr-miR396* family members

**Fig. 1:**
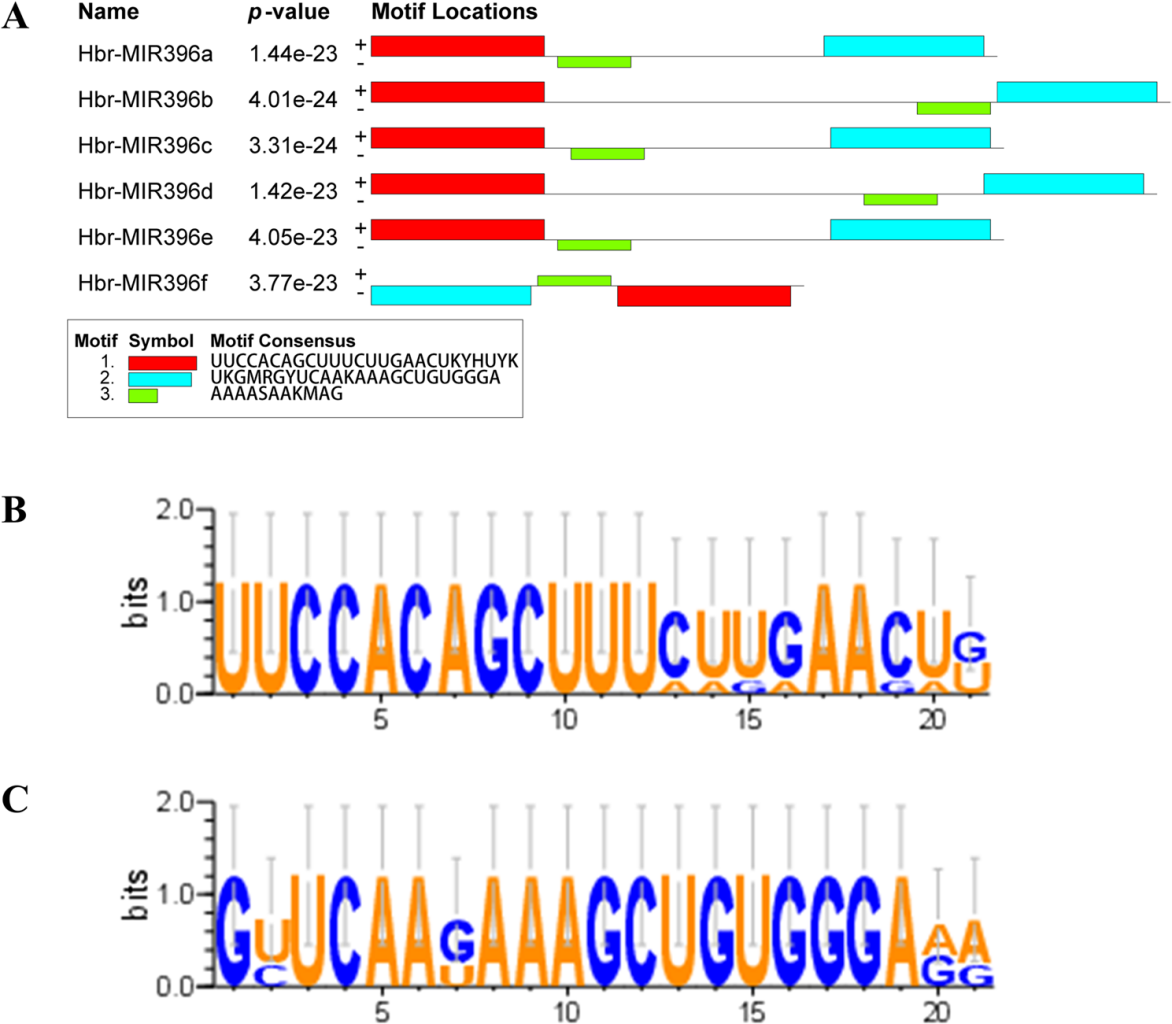
Conservation of sequence features in *Hbr-miR396*. **A** The motif consensus of precursor sequences; (**B**) Mature sequence; (**C**) Star sequence

The *Hbr-miR396* family in the rubber tree genome, consists of six members: *Hbr-miR396*a, b, c, d, e, and f (Table 1, Fig. 1A). The precursor sequences of the six *Hbr-miR396* members range in length from 65 to 120 nucleotides and contain three highly conserved regions. The members of the *miR396* family share similar 5’ and 3’ sequences, while *Hbr-miR396f* shows a reversed situation at both the 5’ and 3’ ends (Fig. 1A).

**Table 1:** Basic information of *Hbr-miR396* family members

miRNA	Chromosome	Gene location	Length	Strand	Mature miRNA	Base number	Star sequence
*Hbr-miR396a*	CM021231.1	7749753_7749660	94	-	UUCCACAGCUUUCUUGAACUU	21	GCUCAAGAAAGCUGUGGGAAA
*Hbr-miR396b*	CM021239.1	73187847_73187966	120	+	UUCCACAGCUUUCUUGAACUG	21	GUUCAAUAAAGCUGUGGGAAG
*Hbr-miR396c*	CM021236.1	80298271_80298365	95	+	UUCCACAGCUUUCUUGAACUU	21	GUUCAAGAAAGCUGUGGGAGA
*Hbr-miR396d*	CM021231.1	7714628_7714745	118	+	UUCCACAGCUUUCUUGAACUG	21	GUUCAAUAAAGCUGUGGGAAG
*Hbr-miR396e*	CM021239.1	73118306_73118212	95	-	UUCCACAGCUUUCUUGAACUU	21	GCUCAAGAAAGCUGUGGGAGA
*Hbr-miR396f*	CM021229.1	93705497_93705435	65	-	UUCCACAGCUUUAAGAAAGAG	21	GUUCAAGAAAGCUGUGGGAGA

The mature *Hbr-miR396* sequences are derived from the highly conserved nucleotides at positions 1–21 of the precursor sequence. *Hbr-miR396a*, *Hbr-miR396c*, and *Hbr-miR396e* share the same mature sequence, while *Hbr-miR396b* and *Hbr-miR396d* have identical mature sequences. In contrast, the mature sequence of *Hbr-miR396*f shows nucleotide variation compared to other members of the *Hbr-miR396* family (Fig. 1B; Table 1). The star sequences of all six *Hbr-miR396* members consist of 21 nucleotides, which exhibit a high degree of conservation (Fig. 1C; Table 1).

Chromosomal localization results show that Hbr-MIR396a and Hbr-MIR396d are located on chromosome CM021231.1, while Hbr-MIR396e and Hbr-MIR396b are found on chromosome CM021239.1. In contrast, Hbr-MIR396f and Hbr-MIR396c are located on chromosomes CM021229.1 and CM021236.1, respectively (Supplementary Fig. 1, Table 1).

### Analysis of the precursor sequence characteristics of the *Hbr-miR396* family members

**Fig. 2:**
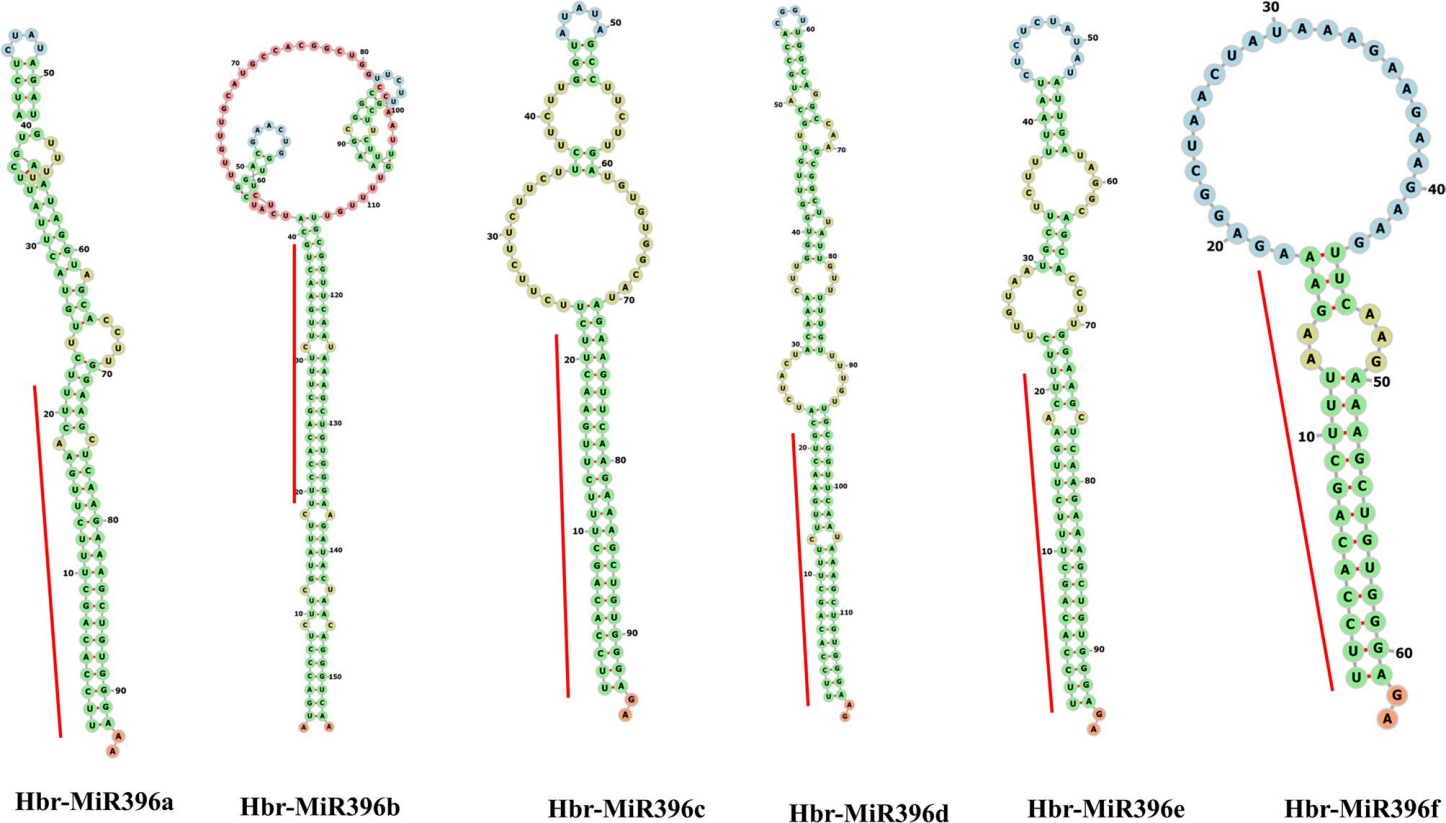
Secondary structure of the precursor sequence of the Hbr-MIR396 family members. Different colors represent different structures. The position of mature *Hbr-miR396* is marked with a red line

The precursor sequences of the six *Hbr-miR396* family members vary in length. The sequence of Hbr-MIR396b is the longest (120 bp), while that of Hbr-MIR396f is the shortest (65 bp). Secondary structure predictions showed that all the precursor sequences could form the stem-loop structures, which are commonly called hairpin structures. All the mature *Hbr-miR396* family members are located on the 5’ arm of their respective precursors (Fig. 2; Table 1).

### Phylogenetic analysis of the *Hbr-miR396* family members

**Fig. 3:**
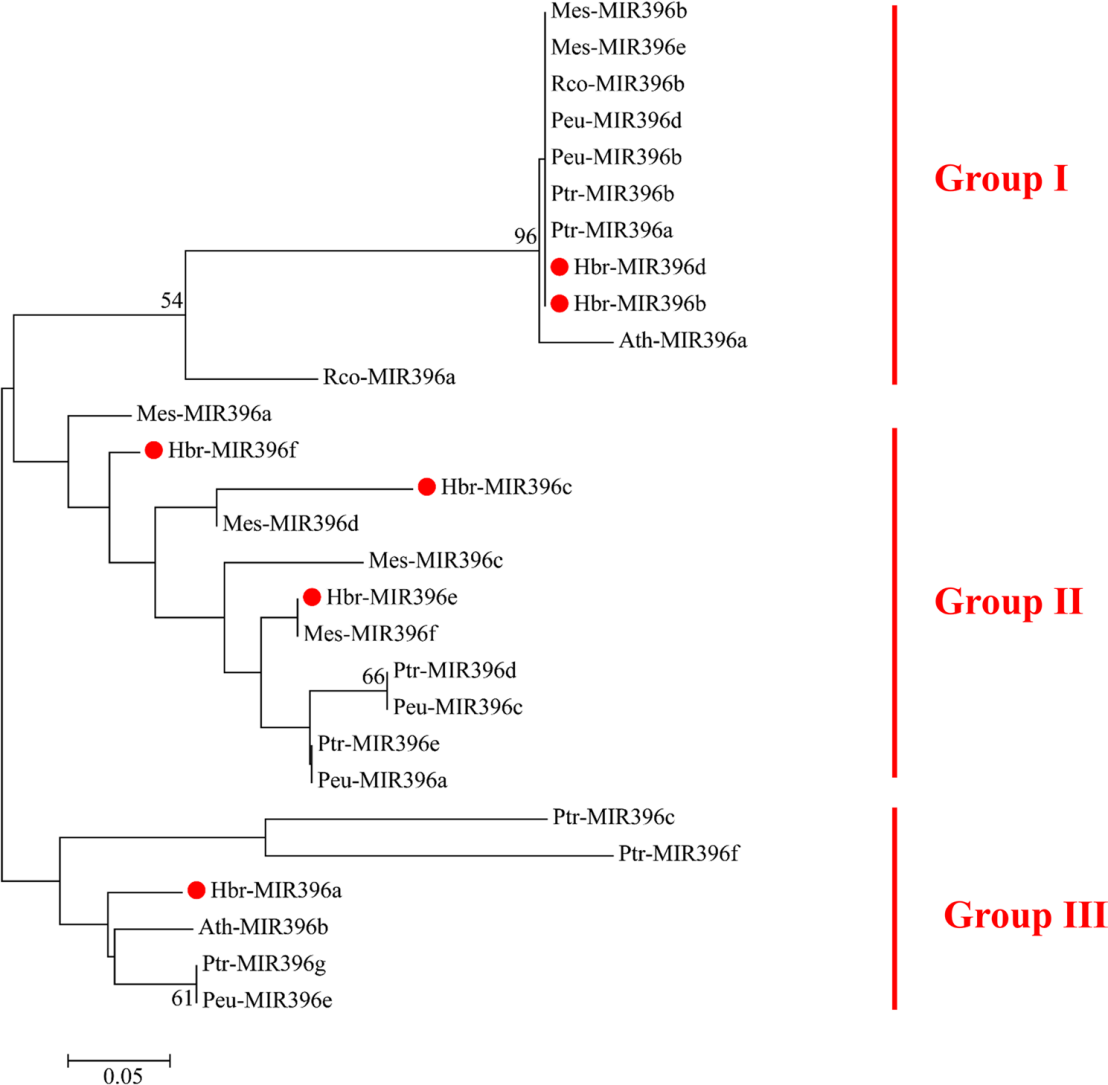
Phylogenetic analysis of the Hbr-MIR396 family members. The phylogenetic tree was generated using the neighbor-joining (NJ) method with a bootstrap value of 1000. Abbreviation Ptr, Peu, Rco, Mes, Ath and Hbr represent *Populus trichocarpa*, *Populus euphratica*, *Ricinus communis*, *Manihot esculenta*, *Arabidopsis thaliana* and *Hevea brasiliensis*. The family members of Hbr-MIR396 in rubber tree are marked with red dots. Group classification (Group I-III) is based on the the clustering of the nucleic acid sequences of MIR396. The scale bar 0.05 mean the degree of variation

Phylogenetic analysis of the precursor sequences revealed that the *miR396* family members of *P. tomentosa*, *P. euphratica*, *R. communis*, *M. esculenta*, and *A. thaliana* could be divided into three groups (Groups I–III). Hbr-MIR396d and Hbr-MIR396b cluster within Group I, while Hbr-MIR396f, Hbr-MIR396c and Hbr-MIR396e cluster within Group II. Compared to *P. tomentosa*, *P. euphratica*, and *A. thaliana*, the rubber tree’s Hbr-MIR396 family has a closer phylogenetic relationship with that of *M. esculenta*. This is primarily reflected in the clustering patterns, where the rubber tree MIRR396 family members, Hbr-MIR396d and Hbr-MIR396b, group together with *M. esculenta* miRNAs Mes-MIR396b and Mes-MIR396e. Hbr-MIR396f clusters with Mes-MIR396a and Hbr-MIR396e groups with Mes-MIR396f (Fig. 3).

### Transcript accumulation analysis of the *Hbr-miR396* family members

Analysis of the levels of transcript accumulation of the Hbr-MIR396 family members in the mature and young leaves of the rubber tree showed that Hbr-MIR396b and Hbr-MIR396d are highly expressed in mature leaves; their levels being the highest compared to the other family members. In contrast, Hbr-MIR396f, Hbr-MIR396c, Hbr-MIR396a, and Hbr-MIR396e were highly expressed in young leaves (Supplementary Fig. 2).

### Prediction and physicochemical characterization of the GRF genes targeted by *Hbr-miR396*

Previous studies have shown that *miR396* primarily influences the growth and development of plants by mediating the GRF regulatory module. Therefore, this study focused on systematically analyzing the predicted GRF genes that could be targeted by *Hbr-miR396*. We found that *Hbr-miR396* targets 8 members of the HbrGRF family, and that the target sites are all located in the exons. The protein sequences of these 8 HbrGRFs range from 232 to 596 amino acids in length, with theoretical isoelectric points (pI) ranging from 7.75 to 9.43 and molecular weights ranging from 25.51 to 67.01 kDa. Prediction of their subcellular localization indicates that all the 8 HbrGRFs may be localized in the nucleus. HbrGRF6 was also predicted to localize in the chloroplast (Table 2).

**Table 2:** Prediction of *Hbr-miR396* target *GRF *genes in *Hevea brasiliensis*

ID	Chromosomal position	Predicted location(s)	Accession ID	Rename	Target positions	pI	MV (kDa)	Length of amino acid
GH714_038451	CM021233.1:3493583–3495707(-)	Nucleus.	KAF2289747.1	HbrGRF 1	Exon	8.6	43.90	406
GH714_009637	CM021226.1:30571269–30572533(-)	Nucleus.	KAF2298060.1	HbrGRF 2	Exon	8.09	37.21	337
GH714_029237	CM021226.1:10029842–10030725(+)	Nucleus.	KAF2298939.1	HbrGRF 3	Exon	8.88	25.51	232
GH714_018771	CM021229.1:97441211–97442644(+)	Nucleus.	KAF2300973.1	HbrGRF 4	Exon	9.43	35.27	316
GH714_021043	CM021234.1:5572484–5577253(+)	Nucleus.	KAF2306744.1	HbrGRF 5	Exon	8.24	35.23	316
GH714_021081	CM021234.1:5505693–5526161(+)	Chloroplast. Nucleus.	KAF2306746.1	HbrGRF 6	Exon	9.07	67.01	596
GH714_030052	CM021234.1:100685805–100687891(+)	Nucleus.	KAF2307599.1	HbrGRF 7	Exon	7.75	41.24	382
GH714_010786	CM021230.1:13456855–13459659(+)	Nucleus.	KAF2324226.1	HbrGRF 8	Exon	8.92	52.02	476

### Gene structure and expression analysis of HbrGRFs

Using the MEME online software prediction platform, the conserved protein sequences of the GRF genes targeted by *Hbr-miR396* were analyzed. A total of 10 motifs were identified, and distributed as following: HbrGRF1 and HbrGRF7 all contained Motif 7, Motif 2, Motif 1, Motif 8, Motif 4, Motif 10 and Motif 6; HbrGRF5 and HbrGRF6 both contained Motif 2, Motif 9, Motif 1, Motif 3, Motif 5, Motif 4 and Motif 10; whereas HbrGRF2 and HbrGRF4 only contained Motif 2 and Motif 1.

**Fig. 4:**
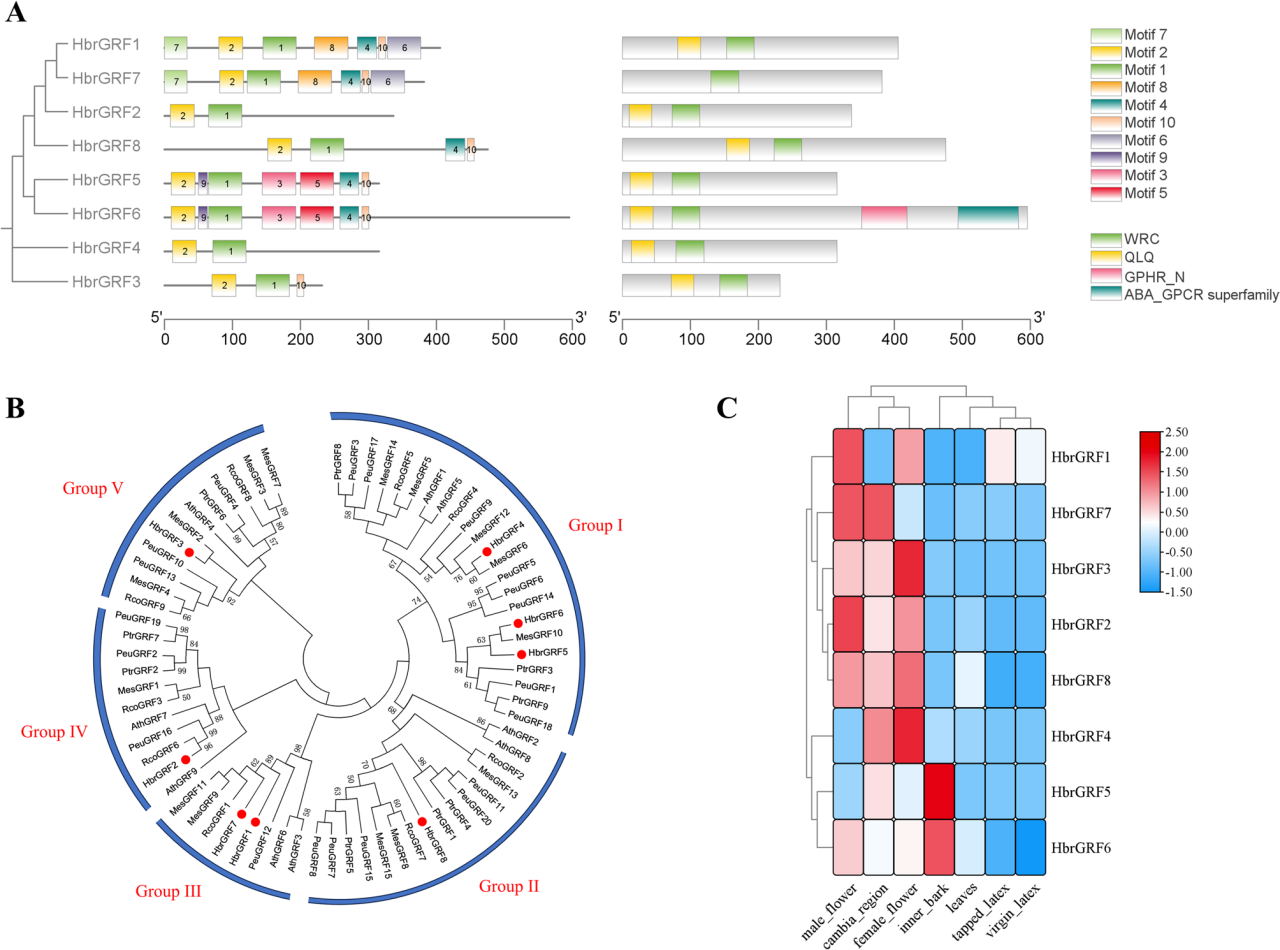
Prediction and sequence features of the target GRF genes of *Hbr-miR396*. **A** Phylogenetic tree, motif distribution and conserved functional domain of the HbrGRF genes family. **B** Phylogenetic tree analysis of rubber tree GRF family members compared with GRF family members from other species. The phylogenetic tree was generated using the neighbor-joining (NJ) method with a bootstrap value of 1000. The family members of GRF in rubber tree are marked with red dots. **C** Expression pattern of HbrGRFs in different tissues. Red and blue boxes indicate high and low of gene expression, respectively. The color key represents normalized expression values (FPKM) of the genes

Using the CDD (Conserved Domain Database) online tool, the conserved domains in the GRF target genes were predicted. A total of four conserved domains were identified. All HbrGRF proteins contained the complete conserved domains at the N-terminus, including the WRC (Motif 1) and QLQ (Motif 2) domains. Additionally, the QLQ domain was located before the WRC domain in these proteins. Moreover, HbrGRF7 also contained the GPHR_N and ABA_GPCR domains (Fig. 4A).

The GRF families in *P. trichocarpa*, *P. euphratica*, *R. communis*, *M. esculenta*, and *A. thaliana* consist of 9, 20, 9, 15, and 9 members, respectively. Using the protein sequences of these GRF genes and the 8 GRF genes from *H. brasiliensis*, a phylogenetic tree was constructed to analyze the evolutionary relationships among the GRF gene families. These GRF genes were clustered into 5 groups (Group I-V). The GRFs from *H. brasiliensis*, *P. trichocarpa*, *P. euphratica*, *R. communis*, *M. esculenta*, and *A. thaliana* were distributed across all the groups, indicating that the GRF genes in these plants may share a similar evolutionary trajectory (Fig. 4B).

*miR396* influences plant growth and development by regulating the expression of its target GRF genes [30]. To investigate the expression patterns of HbrGRFs, transcriptome data from seven different rubber tree tissue types (cambia region, female flower, inner bark, leaves, male flower, tapped latex, and virgin latex) were analyzed. The results revealed that the expression patterns of HbrGRFs exhibited significant tissue specificity. These genes were mainly expressed in tissues with highly active cells, such as the cambia region, female flowers, and male flowers (Fig. 4C).

### Experimental validation of the interaction between *Hbr-miR396b* and HbrGRF3

**Fig. 5:**
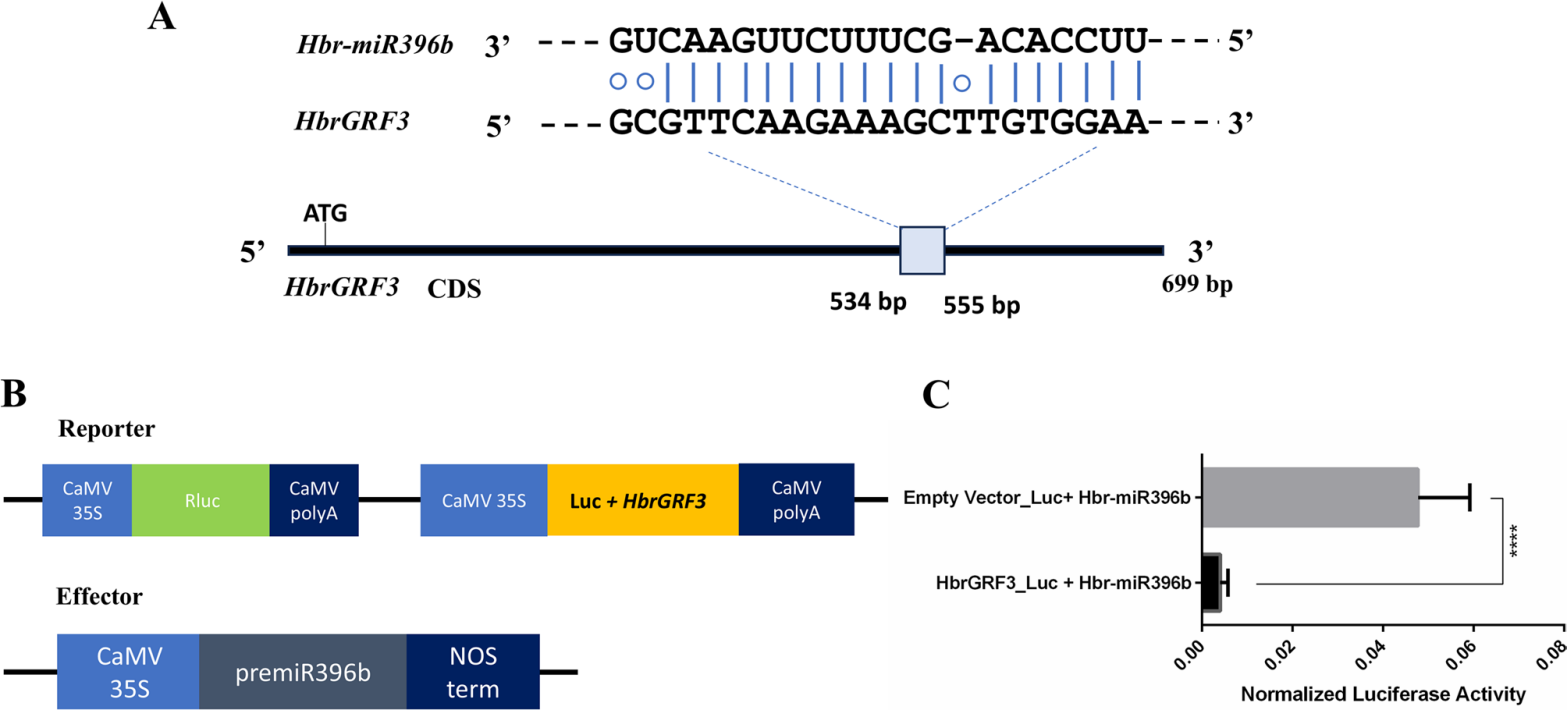
Validation of targeting of HbrGRF3 by *Hbr-miR396*b. **A **Schematic diagrams show how HbrGRF3 is targeted by *Hbr-miR396*b. **B** Schematic diagrams of the gene constructs. **C** Luc analysis confirmed the regulatory relationship between *Hbr-miR396*b and HbrGRF3

Through bioinformatics analysis, the full CDS sequence of HbrGRF3 is comprised of 699 bp, and *Hbr-miR396b* targets the HbrGRF3 sequence from 534 to 555 bp, where a total of 19 complementary bases is observed (Fig. 5A).

We selected the 534–555 bp sequence of HbrGRF3 and cloned it in the pGreen II 0800-miRNA LUC vector. This created a recombinant luciferase fragment containing the *Hbr-miR396b* cleavage sequence, which was named HbrGRF3-Luc as the ‘reporter’. The *Hbr-miR396b* precursor sequence was cloned into the pBI121 vector as the ‘effector’ (Fig. 5B). The pGreen II 0800-LUC empty vector was used as a positive control. Both constructs were transformed into *A. tumefaciens* (GV1301), which was then infiltrated into tobacco leaves to establish a dual-luciferase reporter system. The Luc/Ren fluorescence ratio was measured to determine the mode of action of *Hbr-miR396b* on HbrGRF3. Compared to the control group (the co-expression of pGreen II 0800-LUC empty vector and *Hbr-miR396b*), the experimental group (HbrGRF3-Luc and *Hbr-miR396*b) exhibited a significant reduction in the Luc/Ren fluorescence expression (Fig. 5C).

In addition, the qRT-PCR analysis of the tissue-specific expression of *Hbr-miR396b* and HbrGRF3 in tissue-cultured rubber seedlings showed that *Hbr-miR396b* had the lowest transcript level in the stem, while its target gene HbrGRF3 exhibited the hightest expression level in the strem tissue. These results indicates that *Hbr-miR396b* and HbrGRF3 exhibit different tissue-specific expression patterns (supplementary Fig. 3).

## Discussion

In recent years, miRNAs have garnered significant attention as newly discovered gene regulatory factors and have rapidly become a research hotspot in the field of life sciences. They target mRNAs through complementary base-pairing interactions, leading to transcript degradation or inhibition of corresponding genes expression, and thus, they play key roles in regulating various biological processes. With the continuous improvement of plant genome databases in recent years, research in this area has also increased. Among the miRNAs, *miR396* is a widely conserved family, and is a crucial component of plants [30, 31]. In this study, bioinformatics methods were employed to analyze the characteristics and functions of *Hbr-miR396* family members.

A large number of studies have shown that *miR396* regulates the expression levels of its target genes, the GRFs. Thereby, it modulates plant cell division and differentiation. It plays an important role in various biological processes, such as the development of plant floral organs [10, 12], root development [9, 32], leaf and fruit development [33], and responses to both biotic and abiotic stresses [22, 34]. Currently, research on *miR396* has mainly focused on model plants like rice, *Arabidopsis*, and tobacco, while studies in rubber trees are relatively limited. In this study, the results of the evolutionary analysis of the *Hbr-miR396* family indicate that *miR396* is conserved across species. This study found that the *Hbr-miR396* family in rubber trees consists of 3 mature and 6 precursor sequences. Different precursor sequences could produce identical mature sequences. Except for *Hbr-miR396b*, each mature *Hbr-miR396* originates from the highly conserved 1–21 positions at the 5’ end of the corresponding precursor sequence. Apart from *Hbr-miR396f*, the mature *Hbr-miR396* sequences are highly similar to each other, with only 1 or no base differences among them.

The *miR396* family exhibits a high degree of evolutionary conservation across species [17]. The results of this study indicate that the precursor sequences of *miR396* in rubber trees are more closely related to cassava. This indicates that the evolutionary relationship of the *miR396* gene family is consistent with the phylogenetic relationships of the species themselves. Tissue specific expression analysis revealed that Hbr-MIR396b and Hbr-MIR396d are highly expressed in the mature leaves, while Hbr-MIR396f, Hbr-MIR396c, Hbr-MIR396a, and Hbr-MIR396e are highly expressed in young leaves. This indicates that different members of the *miR396* family may play distinct roles at different stages of leaf development. This differential expression is likely closely associated with the physiological functions and regulatory mechanisms of the leaves at different developmental stages. This could be mediated by *miR396* through modulation of GRFs.

GRFs are plant-specific transcription factors that play a critical role at various stages of plant growth and development. The N-terminus of the GRF proteins typically contains two conserved functional domains, QLQ and WRC [30]. In this study, eight of the *Hbr-miR396*-targeted GRFs exhibited complete conservation of the domains, WRC (Motif 1) and QLQ (Motif 2), where the QLQ domain was positioned before the WRC domain. It has been reported that *miR396* cleaves the conserved WRC domain sequence of GRF genes, which negatively regulates the GRF gene expression [13]. The motif prediction for HbrGRFs show that the 8 HbrGRF proteins possess varying numbers of motifs, which indicates that they may perform different functions in regulating the growth and development of rubber trees.

*Hbr-miR396* may primarily influence the growth and development processes by targeting HbrGRFs and by regulating their expression levels. Therefore, analyzing the expression patterns of HbrGRF genes in rubber trees helped us in understanding the function of the *Hbr-miR396-*HbrGRF module. Expression analysis of HbrGRFs in different tissues revealed a clear tissue-specificity as they were predominantly expressed in the cambia region and the flowers. These findings indicate that HbrGRFs in rubber trees may be mainly involved in regulating the development of floral organs and the growth of the stem. These results are consistent with the previous studies, where members of the GRF gene family are expressed at higher levels in organs or tissues with high developmental activity, and at lower levels in mature tissues or organs [35]. Additionally, in *Brassica napus*, BnGRF regulate the development of floral organs [36].

As post-transcriptional regulators, miRNAs modulate signal transduction, stress responses, and hormone biosynthesis by either degrading target genes or inhibiting their translation [37]. The qRT-PCR analysis of tissue-specific expression of *miR396b* and HbrGRF3 in the rubber seedlings showed an inverse correlation between root and stem, which indicated that *Hbr-miR396b* may negatively regulate the expression of HbrGRF3. Although this association is not observed in leaves, leading to differences in the regulatory relationship between *miR396* and GRF, we speculate that this may be a “precise regulatory strategy” formed by plants during long-term evolution. Through “miRNA expression profile differentiation, target gene functional division, and developmental stage adaptation”, the same regulatory module (*miR396*-GRF) achieves “functional adaptation” in different organs. Moreover, through bioinformatics analysis, we have found that *miR396b* targets HbrGRF3 at the 534–555 bp position. To further validate this prediction, we conducted a dual-luciferase reporter assay, which showed that *miR396b* strongly inhibited the luciferase expression of HbrGRF3_Luc. In addition, we performed a multiple sequence alignment on the loci of the 8 HbrGRF genes targeted by members of the *miR396* family. The results showed that the target sequences are highly conserved and have a high degree of similarity to the HbrGRF3 gene (Supplementary Fig. 5) which has also been confirmed in soybeans [13]. This may also indicate that other *Hbr-miR396* family members regulate the expression of the target HbrGRF *g*enes in the same manner. This further supports the hypothesis that *Hbr-miR396* likely exerts its regulatory effect on the target HbrGRFs by cleaving its mRNA sequence, thereby inhibiting its expression, and ultimately regulating plant growth and development. Furthermore, the verification of each *Hbr-miR396* member will be a primary focus of our subsequent research, utilizing transient expression systems or VIGS (Virus-Induced Gene Silencing) techniques in the rubber tree to further verify the function of the miR396-GRF module in the native species.

Despite certain limitations in the verification of stable inheritance in the original species, such as gain- or loss-of-function studies in rubber tree, to unequivocally establish the causal relationship between *miR396* and HbrGRF. Indeed, genetic transformation and the generation of stable transgenic lines in *Hevea brasiliensis* remain exceptionally challenging due to its long life cycle, low transformation efficiency, and the recalcitrance of its tissues in culture [38, 39]. We believe that the convergent evidence from heterologous validation, spatiotemporal expression patterns, and evolutionary conservation presents a compelling case for the role of the *miR396b*-HbrGRF3 module in rubber tree development. Our work serves as a crucial foundation and provides valuable genetic resources (gene sequences, expression profiles) for the community. This information will provide potential strategies and directions for rubber treee breeding programs. The identified genes (*Hbr-miR396* and HbrGRFs) serve as direct targets for future genetic engineering or gene editing approaches aimed at modulating growth and development of rubber trees.

## Materials and methods

### Genome-wide identification and sequence analysis of *Hbr-miR396* and its target genes

The stem-loop and the mature sequences of the *Hbr-miR396* family members were downloaded from the PmiREN database (https://www.pmiren.com/) [40]. The base conservation of these sequences was analyzed using WebLogo 3 (https://weblogo.threeplusone.com/) [41]. RNAfold WebServe was employed to predict the secondary stem-loop structures of the *Hbr-miR396* precursors (http://rna.tbi.univie.ac.at/cgi-bin/RNAWebSuite/RNAfold.cgi).

The whole genome sequence and annotation files of the rubber tree were downloaded from the NCBI (National Center for Biotechnology Information) database (http://www.ncbi.nlm.nih.gov/) under BioProject accession number PRJNA587314. The miRNA-precursor sequences were then subjected to a BLAST alignment against the rubber tree genome to determine the gene locus information of the Hbr-MIR396 members. The Gene Location Visualization tool in TBtools was used to construct the chromosome localization map [42].

The psRNATarget tool (http://plantgrn.noble.org/psRNATarget/), with default parameters, was used to predict the target genes of *Hbr-miR396* and to analyze the interaction sites between the *Hbr-miR396* and the candidate target genes. Subsequently, the online tool, PlantTFDB (https://planttfdb.gao-lab.org/), was used to screen for target genes that belong to the GRF gene family for further analysis [43]. Additionally, the ExPASy tool (https://web.expasy.org/protparam/) was used to predict the molecular weight (MW) and isoelectric point (pI) of the candidate target genes’ proteins. The subcellular localization of HbrGRF genes was further predicted with the help of the Cell-PLoc 2.0 (http://www.csbio.sjtu.edu.cn/bioinf/Cell-PLoc-2/) online tool [44].

### Phylogenetic analysis of *Hbr-miR396* and its target HbrGRF genes

The precursor stem-loop sequences of *miR396* for *Populus trichocarpa*, *Populus euphratica*, *Ricinus communis*, *Manihot esculenta*, and *A. thaliana* were downloaded from the PmiREN database. The GRF protein sequences for *P. trichocarpa*, *P. euphratica*, *R. communis*, *M. esculenta*, and *A. thaliana* were downloaded from the PlantTFDB database.

The precursor sequences of *miR396* and the GRF protein sequences were aligned with the help of ClustalW [45] with default parameters for multiple sequence alignment. A phylogenetic tree was then constructed with the help of the MEGA v7.0 software, using the neighbor-joining (NJ) method with a bootstrap value of 1000 [46].

### Sequence and structural analysis of HbrGRF genes family targeted by *Hbr-miR396*

The conserved motifs of HbrGRFs were analyzed with the help of the Multiple Expectation Maximization Algorithm for motif prediction (MEME, http://meme-suite.org/). Prediction of the conserved domains of the HbrGRF genes, which could be targeted by *Hbr-miR396*, was performed by using the CDD tool (https://www.ncbi.nlm.nih.gov/Structure/cdd) [47]. Subsequently, the identified motifs and gene structures were further visualized with the help of the TBtools.

### Expression analysis of *Hbr-miR396* and target HbrGRF genes

The expression data of the *Hbr-miR396* family for the young and the mature leaves were downloaded from the PmiREN database for the purpose of differential expression analysis. Transcriptome data of *H. brasiliensis* ‘Reyan 8–79’ (PRJCA004986) were obtained from the National Genomics Data Center (NGDC, https://ngdc.cncb.ac.cn/) [48]. The raw data in Fastq format were first processed using fastp for read trimming, data filtering, and quality control. Clean reads were obtained by removing reads containing sequencing adapters, reads with poly-N, and low-quality reads. These clean reads were then aligned to the reference genome sequence using HISAT2. The gene expression levels were evaluated using FPKM values, and this step was performed using Stringtie2.1.2. This data was used to analyze the differential expression of the *Hbr-miR396* target genes, HbrGRFs, in the seven tissue types: cambium region, female flower, inner bark, male flower, tapped latex, leaves and virgin latex. Transcript levels of *Hbr-miR396* and HbrGRFs were estimated with the FPKM values and scale method was normalized, and heatmaps were generated by using the TBtools.

### Dual-luciferase assay

To verify the miRNA targets, the binding-site sequences of HbrGRF3 were inserted between XbaI and EcoRI restriction site of the pGreen II 0800-miRNA LUC vector, downstream of the cauliflower mosaic virus (CaMV) 35 S promoter, and were used as reporters. The precursor sequence of Hbr-MIR396b was amplified from the cDNA samples of rubber tree (Renyan 7–33−97) [49]. These PCR products were cloned into between XbaI and SacI restriction site a binary plant expression pBI121 vector to generate 35 S::*miR396b*, and used as an effector. The plasmids were transferred into *A. tumefaciens* GV1301 and co-infiltrated into the *N. benthamiana* leaves. The luciferase activities were measured with the help of the Dual-Luciferase Reporter Assay System (Promega, Madison, WI, USA), according to the manufacturer’s instructions.

### RNA extraction and quantitative reverse transcription PCR (qRT-PCR)

The rubber tissue culture seedlings that are 1 year old were selected for sampling of roots, stems, and leaves. Total RNA was extracted by using the TRIzol reagent (Invitrogen), which was followed by a DNase treatment to remove genomic DNA contamination. Complementary DNA (cDNA) was synthesized by using the PrimeScript™ RT reagent kit and gDNA Eraser (TaKaRa), according to the manufacturer’s instructions. The qRT-PCR was performed with the help of SYBR Premix ExTaq™ (TaKaRa) on a thermal cycler. PCR amplification was performed at a thermal cycler heat block and incubated at 95 ◦C for 30 s, followed by 40 cycles of 95 ◦C for 5 s and 60 ◦C for 30 s. The Ubiquitin genes served as internal controls for normalization. Mature *Hbr-miR396b* was reverse transcribed by using a specific stem-loop reverse transcription primer, and analyzed by stem-loop qRT-PCR detection Kit (Aidlab, Beijing, China). PCR amplification was performed at a thermal cycler heat block and incubated at 94 ◦C for 3 min, followed by 40 cycles of 94 ◦C for 10 s and 60 ◦C for 32 s. The U6 genes served as internal reference gene for normalization.

For melting curve analysis, samples were denatured at 95 ◦C for 30 s, then cooled to 65 ◦C for 30s. Fluorescence signals were collected continuously from 65 ◦C to 95 ◦C at 0.5 ◦C per 5 s. The expression levels of the *Hbr-miR396b* and HbrGRF3 gene were calculated using the 2^–△△Ct^ method against the internal controls, with the expression levels of *Hbr-miR396b* and HbrGRF3 in roots are set to 1. Three technical replicates per sample were analyzed to ensure the reliability. The asterisks indicated the significant differences based on Student’s t-test (*, *P* < 0.05; **, *P* < 0.01;***, *P* < 0.001). The gene-specific primers that were used to determine expression levels are listed in Supplementary Tables 1 and Melt Curve and Amplification plots of *Hbr-miR396b* and HbrGRF3 are presented in Supplementary Fig. 4.

## Conclusions

In this study, we have identified 6 *Hbr-miR396* family members from the rubber tree genome, which could target 8 HbrGRF genes. Through molecular validation, we also have confirmed the interaction between *Hbr-miR396b* and HbrGRF3. As post-transcriptional regulators, the *Hbr-miR396* family members could primarily regulate plant growth and development by targeting GRF genes. Most HbrGRF genes exhibit higher expression levels in the actively developing tissues and organs, such as the cambia region and flowers. Therefore, we hypothesize that *Hbr-miR396* may potentially regulate growth and floral organ development in rubber trees by targeting HbrGRFs. This study is the first comprehensive identification of the *Hbr-miR396*-HbrGRFs module in the rubber trees. This research provides a foundation for further elucidating the molecular mechanisms of the *Hbr-miR396*-HbrGRFs module in the growth and development of rubber trees.

## Supplementary Information

Additional file 1: Supplementary Figure 1. Chromosome location of Hbr-MIR396 family members. Supplementary Figure 2. The specific expression of Hbr-MIR396 members in mature and young leaves tissue.The color key represents normalized expression values (FPKM) of the Hbr-MIR396 members. Supplementary Figure 3. The relative transcript levels of *Hbr-miR396b* and HbrGRF3 in different tissues (root, stem, and leaf) of rubber tree. Supplementary Figure 4. Melt Curve and Amplification plots of *Hbr-miR396b* (A and B)HbrGRF3 (C and D) are presented. Supplementary Figure 5. Sequence alignment of the* Hbr-miR39*6 complementary sequences with the target sites in HbrGRF genes. Supplementary Table 1. Oligonucleotide primers used for qRT-PCR. Supplementary Table 2. The detailed targeting location and sequence information of HbrGRFs targeted by *Hbr-miR396* members. Supplementary Table 3. Summary of sequence information for GRF genes from other species used in phylogenetic tree construction.

## Data Availability

All data generated or analyzed during this study are included in this paper and its supplementary information files. The whole genome sequence and annotation files of H. brasiliensis were downloaded from the NCBI (National Center for Biotechnology Information) database (http://www.ncbi.nlm.nih.gov/) under BioProject accession number PRJNA587314. Additionally, the RNA-seq data of *H. brasiliensis* (PRJCA004986) were obtained from the National Genomics Data Center (NGDC, https://ngdc.cncb.ac.cn/).
