# Supplementary material for: Genome-wide identification and analysis of miR396 family members and their target GRF genes in rubber tree (Hevea brasiliensis)
Source: BMC Genomics. 2025 Oct 31;26:985. doi: 10.1186/s12864-025-12156-x (PMC12577359; doi:10.1186/s12864-025-12156-x)
Supplement: Supplementary file 1 — Additional file 1: Supplementary Figure 1. Chromosome location of Hbr-MIR396 family members. Supplementary Figure 2. The specific expression of Hbr-MIR396 members in mature and young leaves tissue.The color key represents normalized expression values (FPKM) of the Hbr-MIR396 members. Supplementary Figure 3. The relative transcript levels of Hbr-miR396b and HbrGRF3 in different tissues (root, stem, and leaf) of rubber tree. Supplementary Figure 4. Melt Curve and Amplification plots of Hbr-miR396b (A and B)HbrGRF3 (C and D) are presented. Supplementary Figure 5. Sequence alignment of the Hbr-miR396 complementary sequences with the target sites in HbrGRF genes. Supplementary Table 1. Oligonucleotide primers used for qRT-PCR. Supplementary Table 2. The detailed targeting location and sequence information of HbrGRFs targeted by Hbr-miR396 members. Supplementary Table 3. Summary of sequence information for GRF genes from other species used in phylogenetic tree construction. [file 12864_2025_12156_MOESM1_ESM.pdf]

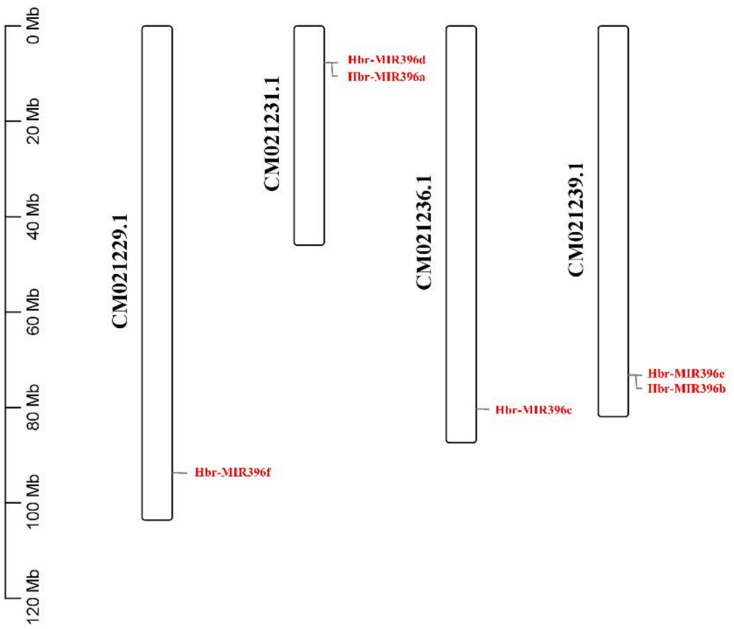

Supplementary Figure 1. Chromosome location of Hbr-MIR396 family members.

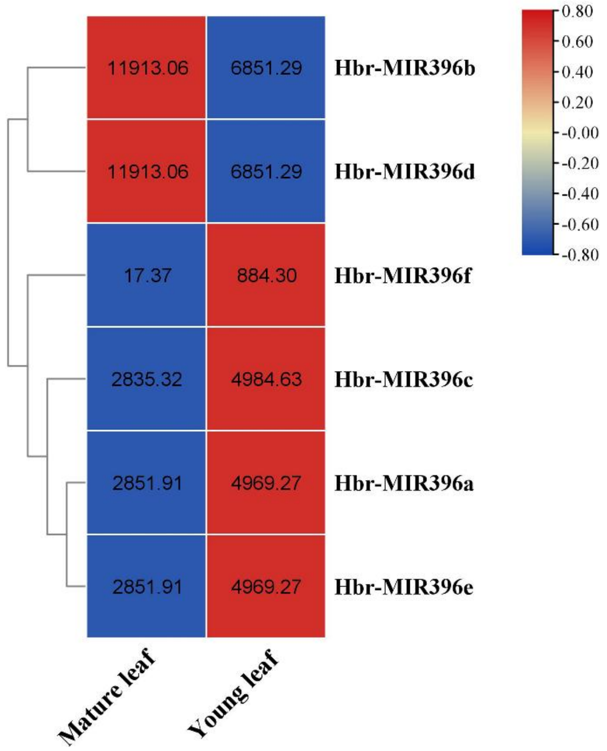

Supplementary Figure 2. The specific expression of Hbr-MIR396 members in mature and young leaves tissue.The color key represents normalized expression values (FPKM) of the Hbr-MIR396 members.

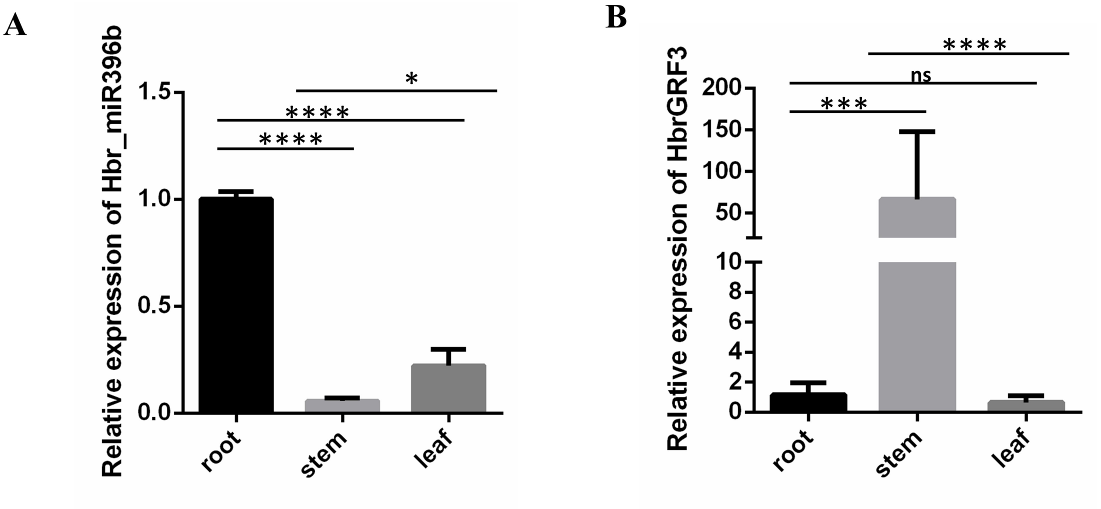

Supplementary Figure 3. The relative transcript levels of *Hbr-miR396b* (A) and *Hbr-GRF3* (*B*) in different tissues (root, stem, and leaves) of rubber tree.

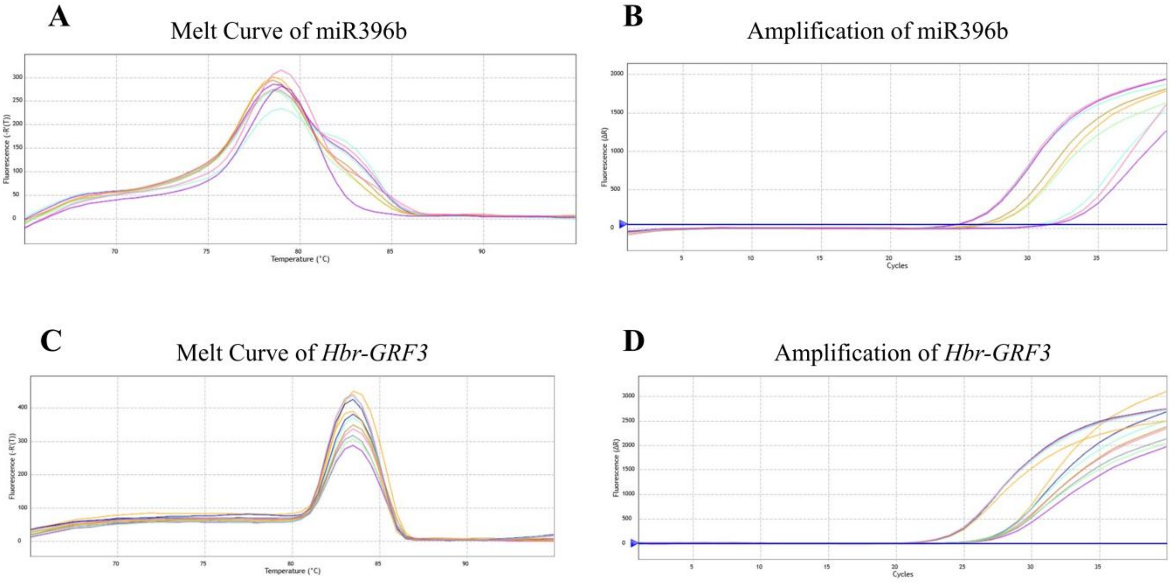

Supplementary Figure 4. Melt Curve and Amplification plots of miR396b (A and B) Hbr-GRF3 (C and D) are presented.

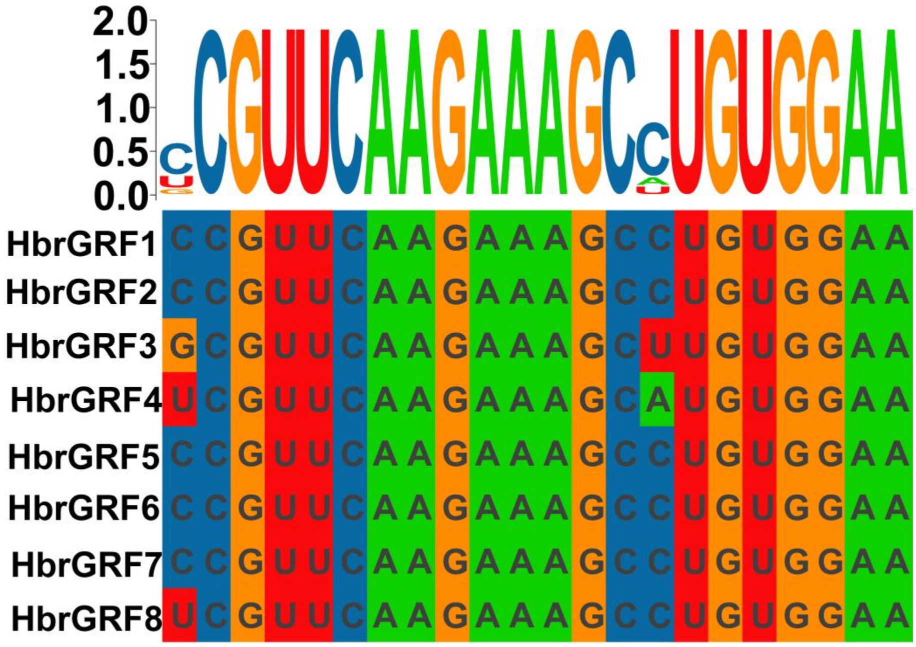

Supplementary Figure 5. Sequence alignment of the *Hbr-miR396* complementary sequences with the target sites in *HbrGRF* genes

Supplementary Table 1. Oligonucleotide primers used for qPCR

Primer name	Primer sequence (5' to 3')
F_HbrGRF3	GGTTAGGACTTGCGCTTGGA
R_HbrGRF3	AGATTTTGTGCAGTCGGGGA
F-Hbr-miR396b	GCGTTCCACAGCTTTCTTGAACTG
F-Ubiquitin	ATGCAGCACCGGGAATAAGT
R-Ubiquitin	GGTCATCAGGGTTTGGAGCA
F-U6	CATCCGATAAAATTGGAACG
R-U6	GATTTGTGCGTGTCATCCTT

Supplementary Table 2. The detailed targeting location and sequence information of *GRFs* targeted by *Hbr-miR396* members.

miRNA_Acc.	Target_Acc.	Target_start	Target_end	miRNA_aligned_fragment	alignment	Target_aligned_fragment	Inhibition
Hbr-miR396a	GH714_030052	1068	1089	UUCCACA-GCUUUCUUGAACUU	:::::::::::: :::::::	CCGUUCAAGAAAGCCUGUGGAA	Cleavage
Hbr-miR396a	GH714_009637	391	412	UUCCACA-GCUUUCUUGAACUU	:::::::::::: :::::::	CCGUUCAAGAAAGCCUGUGGAA	Cleavage
Hbr-miR396a	GH714_021043	1365	1386	UUCCACA-GCUUUCUUGAACUU	:::::::::::: :::::::	CCGUUCAAGAAAGCCUGUGGAA	Cleavage
Hbr-miR396a	GH714_021081	1367	1388	UUCCACA-GCUUUCUUGAACUU	:::::::::::: :::::::	CCGUUCAAGAAAGCCUGUGGAA	Cleavage
Hbr-miR396a	GH714_010786	1941	1962	UUCCACA-GCUUUCUUGAACUU	:::::::::::: :::::::	UCGUUCAAGAAAGCCUGUGGAA	Cleavage
Hbr-miR396a	GH714_018771	449	470	UUCCACA-GCUUUCUUGAACUU	:::::::::::: :::::::	UCGUUCAAGAAAGCAUGUGGAA	Cleavage
Hbr-miR396a	GH714_029237	719	740	UUCCAC-AGCUUUCUUGAACUU	::::::::::::: ::::::	GCGUUCAAGAAAGCUUGUGGAA	Cleavage
Hbr-miR396a	GH714_038451	1080	1101	UUCCACA-GCUUUCUUGAACUU	:::::::::::: :::::::	CCGUUCAAGAAAGCCUGUGGAA	Cleavage
Hbr-miR396b	GH714_038451	1080	1101	UUCCACA-GCUUUCUUGAACUG	: :::::::::::: :::::::	CCGUUCAAGAAAGCCUGUGGAA	Cleavage
Hbr-miR396b	GH714_009637	391	412	UUCCACA-GCUUUCUUGAACUG	: :::::::::::: :::::::	CCGUUCAAGAAAGCCUGUGGAA	Cleavage
Hbr-miR396b	GH714_030052	1068	1089	UUCCACA-GCUUUCUUGAACUG	: :::::::::::: :::::::	CCGUUCAAGAAAGCCUGUGGAA	Cleavage
Hbr-miR396b	GH714_021043	1365	1386	UUCCACA-GCUUUCUUGAACUG	: :::::::::::: :::::::	CCGUUCAAGAAAGCCUGUGGAA	Cleavage
Hbr-miR396b	GH714_021081	1367	1388	UUCCACA-GCUUUCUUGAACUG	: :::::::::::: :::::::	CCGUUCAAGAAAGCCUGUGGAA	Cleavage
Hbr-miR396b	GH714_010786	1941	1962	UUCCACA-GCUUUCUUGAACUG	:::::::::::: :::::::	UCGUUCAAGAAAGCCUGUGGAA	Cleavage
Hbr-miR396b	GH714_018771	449	470	UUCCACA-GCUUUCUUGAACUG	:::::::::::: :::::::	UCGUUCAAGAAAGCAUGUGGAA	Cleavage
Hbr-miR396b	GH714_029237	719	740	UUCCAC-AGCUUUCUUGAACUG	::::::::::::: ::::::	GCGUUCAAGAAAGCUUGUGGAA	Cleavage
Hbr-miR396c	GH714_021043	1365	1386	UUCCACA-GCUUUCUUGAACUU	:::::::::::: :::::::	CCGUUCAAGAAAGCCUGUGGAA	Cleavage
Hbr-miR396c	GH714_010786	1941	1962	UUCCACA-GCUUUCUUGAACUU	:::::::::::: :::::::	UCGUUCAAGAAAGCCUGUGGAA	Cleavage
Hbr-miR396c	GH714_021081	1367	1388	UUCCACA-GCUUUCUUGAACUU	:::::::::::: :::::::	CCGUUCAAGAAAGCCUGUGGAA	Cleavage
Hbr-miR396c	GH714_018771	449	470	UUCCACA-GCUUUCUUGAACUU	:::::::::::: :::::::	UCGUUCAAGAAAGCAUGUGGAA	Cleavage
Hbr-miR396c	GH714_038451	1080	1101	UUCCACA-GCUUUCUUGAACUU	:::::::::::: :::::::	CCGUUCAAGAAAGCCUGUGGAA	Cleavage
Hbr-miR396c	GH714_029237	719	740	UUCCAC-AGCUUUCUUGAACUU	::::::::::::: ::::::	GCGUUCAAGAAAGCUUGUGGAA	Cleavage
Hbr-miR396c	GH714_030052	1068	1089	UUCCACA-GCUUUCUUGAACUU	:::::::::::: :::::::	CCGUUCAAGAAAGCCUGUGGAA	Cleavage
Hbr-miR396c	GH714_009637	391	412	UUCCACA-GCUUUCUUGAACUU	:::::::::::: :::::::	CCGUUCAAGAAAGCCUGUGGAA	Cleavage
Hbr-miR396d	GH714_009637	391	412	UUCCACA-GCUUUCUUGAACUG	: :::::::::::: :::::::	CCGUUCAAGAAAGCCUGUGGAA	Cleavage
Hbr-miR396d	GH714_038451	1080	1101	UUCCACA-GCUUUCUUGAACUG	: :::::::::::: :::::::	CCGUUCAAGAAAGCCUGUGGAA	Cleavage
Hbr-miR396d	GH714_021043	1365	1386	UUCCACA-GCUUUCUUGAACUG	: :::::::::::: :::::::	CCGUUCAAGAAAGCCUGUGGAA	Cleavage
Hbr-miR396d	GH714_021081	1367	1388	UUCCACA-GCUUUCUUGAACUG	: :::::::::::: :::::::	CCGUUCAAGAAAGCCUGUGGAA	Cleavage
Hbr-miR396d	GH714_030052	1068	1089	UUCCACA-GCUUUCUUGAACUG	: :::::::::::: :::::::	CCGUUCAAGAAAGCCUGUGGAA	Cleavage

miRNA_Acc.	Target_Acc.	Target_start	Target_end	miRNA_aligned_fragment	alignment	Target_aligned_fragment	Inhibition
Hbr-miR396d	GH714_010786	1941	1962	UUCCACA-GCUUUCUUGAACUG	:::::::::::: :::::::	UCGUUCAAGAAAGCCUGUGGAA	Cleavage
Hbr-miR396d	GH714_018771	449	470	UUCCACA-GCUUUCUUGAACUG	:::::::::::: :::::::	UCGUUCAAGAAAGCAUGUGGAA	Cleavage
Hbr-miR396d	GH714_029237	719	740	UUCCAC-AGCUUUCUUGAACUG	::::::::::::: ::::::	GCGUUCAAGAAAGCUUGUGGAA	Cleavage
Hbr-miR396e	GH714_018771	449	470	UUCCACA-GCUUUCUUGAACUU	:::::::::::: :::::::	UCGUUCAAGAAAGCAUGUGGAA	Cleavage
Hbr-miR396e	GH714_038451	1080	1101	UUCCACA-GCUUUCUUGAACUU	:::::::::::: :::::::	CCGUUCAAGAAAGCCUGUGGAA	Cleavage
Hbr-miR396e	GH714_021043	1365	1386	UUCCACA-GCUUUCUUGAACUU	:::::::::::: :::::::	CCGUUCAAGAAAGCCUGUGGAA	Cleavage
Hbr-miR396e	GH714_030052	1068	1089	UUCCACA-GCUUUCUUGAACUU	:::::::::::: :::::::	CCGUUCAAGAAAGCCUGUGGAA	Cleavage
Hbr-miR396e	GH714_029237	719	740	UUCCAC-AGCUUUCUUGAACUU	::::::::::::: ::::::	GCGUUCAAGAAAGCUUGUGGAA	Cleavage
Hbr-miR396e	GH714_009637	391	412	UUCCACA-GCUUUCUUGAACUU	:::::::::::: :::::::	CCGUUCAAGAAAGCCUGUGGAA	Cleavage
Hbr-miR396e	GH714_021081	1367	1388	UUCCACA-GCUUUCUUGAACUU	:::::::::::: :::::::	CCGUUCAAGAAAGCCUGUGGAA	Cleavage
Hbr-miR396e	GH714_010786	1941	1962	UUCCACA-GCUUUCUUGAACUU	:::::::::::: :::::::	UCGUUCAAGAAAGCCUGUGGAA	Cleavage
Hbr-miR396b	GH714_021081	11235	11255	UUCCACAGCUUUCUUGAACUG	:: .:::.::.:::::::	UUGUGGGAGAGAGUUGUGGAA	Cleavage
Hbr-miR396d	GH714_021081	11235	11255	UUCCACAGCUUUCUUGAACUG	:: .:::.::.:::::::	UUGUGGGAGAGAGUUGUGGAA	Cleavage

Supplementary Table 3. Summary of sequence information for *GRF* genes from other species used in phylogenetic tree construction.

Gene ID	Species	Rename
Potri.007G007100.1	*Populus_trichocarpa*	PtrGRF1
Potri.012G022600.1	*Populus_trichocarpa*	PtrGRF2
Potri.013G077500.1	*Populus_trichocarpa*	PtrGRF3
Potri.014G007200.1	*Populus_trichocarpa*	PtrGRF4
Potri.014G012800.1	*Populus_trichocarpa*	PtrGRF5
Potri.014G071800.1	*Populus_trichocarpa*	PtrGRF6
Potri.015G006200.1	*Populus_trichocarpa*	PtrGRF7
Potri.018G065400.1	*Populus_trichocarpa*	PtrGRF8
Potri.019G042300.1	*Populus_trichocarpa*	PtrGRF9
CCG001468.1	*Populus_euphratica*	PeuGRF1
CCG003652.1	*Populus_euphratica*	PeuGRF2
CCG006318.1	*Populus_euphratica*	PeuGRF3
CCG010577.1	*Populus_euphratica*	PeuGRF4
CCG014363.1	*Populus_euphratica*	PeuGRF5
CCG014365.1	*Populus_euphratica*	PeuGRF6
CCG015382.1	*Populus_euphratica*	PeuGRF7
CCG016350.1	*Populus_euphratica*	PeuGRF8
CCG021012.1	*Populus_euphratica*	PeuGRF9
CCG021329.1	*Populus_euphratica*	PeuGRF10
CCG022471.1	*Populus_euphratica*	PeuGRF11
CCG023815.1	*Populus_euphratica*	PeuGRF12
CCG024408.1	*Populus_euphratica*	PeuGRF13
CCG024718.1	*Populus_euphratica*	PeuGRF14
CCG027670.1	*Populus_euphratica*	PeuGRF15
CCG029619.1	*Populus_euphratica*	PeuGRF16
CCG031268.1	*Populus_euphratica*	PeuGRF17
CCG033904.1	*Populus_euphratica*	PeuGRF18
CCG034010.1	*Populus_euphratica*	PeuGRF19
CCG011804.2	*Populus_euphratica*	PeuGRF20
27496.m000094	*Ricinus_communis*	RcoGRF1
28976.m000158	*Ricinus_communis*	RcoGRF2
29700.m000761	*Ricinus_communis*	RcoGRF3
29706.m001283	*Ricinus_communis*	RcoGRF4
29739.m003775	*Ricinus_communis*	RcoGRF5
30068.m002588	*Ricinus_communis*	RcoGRF6
30170.m013673	*Ricinus_communis*	RcoGRF7
30174.m008910	*Ricinus_communis*	RcoGRF8
30190.m011066	*Ricinus_communis*	RcoGRF9
Manes.01G041800.1.p	*Manihot_esculenta*	MesGRF1
Manes.01G070800.1.p	*Manihot_esculenta*	MesGRF2
Manes.01G264700.1.p	*Manihot_esculenta*	MesGRF3

Manes.02G031200.1.p	*Manihot_esculenta*	MesGRF4
Manes.03G039500.1.p	*Manihot_esculenta*	MesGRF5
Manes.04G144700.1.p	*Manihot_esculenta*	MesGRF6
Manes.05G043700.1.p	*Manihot_esculenta*	MesGRF7
Manes.05G183900.1.p	*Manihot_esculenta*	MesGRF8
Manes.08G022300.1.p	*Manihot_esculenta*	MesGRF9
**Accession ID**	***Species***	**Rename**
Manes.08G160800.1.p	*Manihot_esculenta*	MesGRF10
Manes.09G059500.1.p	*Manihot_esculenta*	MesGRF11
Manes.11G020200.1.p	*Manihot_esculenta*	MesGRF12
Manes.12G117600.1.p	*Manihot_esculenta*	MesGRF13
Manes.16G096400.1.p	*Manihot_esculenta*	MesGRF14
Manes.18G049600.1.p	*Manihot_esculenta*	MesGRF15
AT2G06200.1	*Arabidopsis_thaliana*	AthGRF1
AT2G22840.1	*Arabidopsis_thaliana*	AthGRF2
AT2G36400.1	*Arabidopsis_thaliana*	AthGRF3
AT2G45480.1	*Arabidopsis_thaliana*	AthGRF4
AT3G13960.1	*Arabidopsis_thaliana*	AthGRF5
AT3G52910.1	*Arabidopsis_thaliana*	AthGRF6
AT4G24150.1	*Arabidopsis_thaliana*	AthGRF7
AT4G37740.1	*Arabidopsis_thaliana*	AthGRF8
AT5G53660.1	*Arabidopsis_thaliana*	AthGRF9
